# Elastic Membrane That Undergoes Mechanical Deformation Enhances Osteoblast Cellular Attachment and Proliferation

**DOI:** 10.1155/2010/947232

**Published:** 2010-06-27

**Authors:** G. K. Toworfe, R. J. Composto, M. H. Lee, P. Ducheyne

**Affiliations:** ^1^Center for Bioactive Materials and Tissue Engineering, Department of Bioengineering, SEAS, University of Pennsylvania, 210S 33rd Street, Philadelphia, PA 19104, USA; ^2^Department of Materials Science and Engineering, SEAS, University of Pennsylvania, 321 LRSM, Walnut Street, Philadelphia, PA 19104, USA; ^3^Department of Bioengineering, SEAS, University of Pennsylvania, 115 Hayden Hall, 210S 33rd Street, Philadelphia, PA 19104, USA

## Abstract

The main objective of this paper was to investigate the effect of transmission of force on bone cells that were attached to a deformable membrane. We functionalized a silastic membrane that measured 0.005 inches thickness and coated it with an extra cellular matrix (ECM) protein, fibronectin (FN). MC3T3-E1 osteoblast-like cells were cultured on the functionalized FN-coated membrane after which cell attachment and proliferation were evaluated. We observed an immediate attachment and proliferation of the bone cells on the functionalized membrane coated with FN, after 24 hours. Upon application of a mechanical force to cells cultured on the functionalized silicone membrane in the form of a dynamic equibiaxial strain, 2% magnitude; at 1-Hz frequency for 2 h, the osteoblast cells elicited slightly elevated phalloidin fluorescence, suggesting that there was reorganization of the cytoskeleton. We concluded from this preliminary data obtained that the engineered surface transduced applied mechanical forces directly to the adherent osteoblast cells via integrin binding tripeptide receptors, present in the FN molecules, resulting in the enhanced cellular attachment and proliferation.

## 1. Introduction

Modified and deformable bioactive substrates can enhance osteoblast adhesion and proliferation. The surface reactivity and interfacial adhesion of such substrates to bone cells can be attributed to the nature/type of surface modifications and functionalized layers attached to the bioactive materials. Bone cells transduce changes in the mechanical environment through adhesion molecules that link them to the extracellular matrix (ECM). The modifications and deformations of substrates are therefore likely to influence the actin cytoskeleton [1]. Very few studies to date, have examined the combined effect of both mechanical stress and materials surface properties on bone cell function. This paper;therefore, investigates the effect of mechanical stimulus and substrate surface characteristics on osteoblasts attachment and proliferation. We hypothesized that the proliferation stage of osteoblastic cells to strain responses *in vitro* could be determined by the combined effect of applied mechanical strain and surface properties of a substrate. This paper will, thus, serve as a fundamental study in understanding mechanotransduction processes in bone cells seeded on modified bioactive substrates. 

### 1.1. Effect of Mechanical Stress on Bone Cells

Bone cells respond to mechanical stimulation and as a result, bone architecture is strongly influenced by mechanical force through a mechanism by which bone cells adapt to mechanical loading. Previous studies that have reported on the responses of bone cells to mechanical stimuli varied widely and therefore lacked consensus on what mechanisms of mechanotransduction were physiologically relevant [2]. Minimal strains have been reported to occur in bone cells as a result of applied mechanical stresses under physiological conditions [3, 4]. Some reported studies have suggested, however that strain rate correlated with bone formation [5, 6]. One study had suggested that mechanical forces were transmitted to cells through the extra cellular matrix [7] and recently published evidence suggested that the ECM-cell surface receptors (the integrins) may act as mechanoreceptors [8, 9]. In one study that focused on the mechanosensitivity of human bone derived cells, the authors demonstrated the frequency and cycle number dependence of the proliferative response of human osteoblast-like cells [10]. That study further surmised that those two effects should not be considered separately since they were interrelated. In a recent study, however, a device was developed that enabled application of sinusoidal micromotions of amplitudes between 5–50 *μ*m and applied loads up to 1000 Pa on cells,* in vitro,* to analyze the bone-implant interface [11]. In another recent and related study [12], some investigators reported that short periods of applied physiological mechanical stresses induced immediate early gene expression and growth in MC3T3-E1 osteoblasts. 

Various techniques have been proposed and used* in vitro* by several investigators to study mechanostimulation of bone cells. Such studies have often used systems that included cell culture with controlled delivery of a mechanical input like hydrostatic pressure, fluid shear stress, and a substrate strain [13]. Apparatuses devised in the laboratory for such studies included a variety of complex systems that featured mechanical inputs of varied degrees of precision and homogeneity although early efforts in cell culture mechanostimulus were of a nonquantitative nature. Glucksmann [14] who pioneered cell mechanostimulus studies utilized several biological-loading models like endosteal cell cultures from embryonic chick tibiae. Quantitation in cell mechanostimulus studies was achieved by the landmark study of Rodan and coworkers [15], which involved preparations of a variety of cells and explants as well as hydrostatic pressurization of suspended bone cells and tensile straining. Techniques for mechanical stimulation of cells that have been employed in the past, included use of hydrostatic pressure, axial compression, longitudinal mechanical straining, out-of-plane systems, and fluid shear stress systems. Special purpose systems were developed to study the combined effect of fluid stress and substrate deformation concurrently [16–18]. Among the various mechanostimulus systems used, there was a strong preference for the longitudinal systems that were designed to effectively apply mechanical deformation to bone cells seeded on deformable membranes. In a most recent study, however, the authors confirmed that although mechanical stress was essential for the survival of cells and the maintenance of tissues, the mechanisms of cellular response to mechanical stress were not fully elucidated due to the diversity of mechanical stresses and mechanosensors [19]. 

In this study, we developed and designed a system that enhanced the attachment and proliferation of bone cells to a deformable membrane. The objectives of this study were to anchor osteoblast cells to a substrate that was chemically functionalized and then modified with protein molecules; and to ascertain whether the combined effect of substrate modifications and applied equibiaxial mechanical strain could cause a change in the cytoskeletal architecture of the adherent bone cells.

## 2. Materials and Methods

Prior to anchorage of cells to the substrate with attached protein molecules, the silicone membrane was functionalized by exposure to ultra violet radiation (UVO). The substrates were then characterized using the established surface characterizations tools such as contact angle goniometry, atomic force microscopy (AFM), and Rutherford backscattering spectroscopy (RBS). Bone cells (MC3T3-E1 osteoblasts) were then seeded onto the biomimetic surface, followed by evaluation of the cell function to determine changes in the cytoskeletal organization of the adherent cells. 

### 2.1. Preparation of Silicone Membranes

Silicone membranes of 0.005 inches thickness (Silastic Q7-4840, Specialty Manufacturing Inc., Saginaw, MI) and 2.5 inches cross-sectional diameter were used as substrates for the studies. Membranes were first functionalized, coated with FN, and then cultured in cells prior to application of mechanical strain.

#### 2.1.1. Functionalization of Silicone Membranes

The silicone membranes were exposed to 10 to 30 min UVO radiation in order to functionalize and oxidize the surfaces. This treatment resulted in the formation of active hydroxyl (-OH) groups on the surface of the membrane, compared to untreated surfaces, which remained hydrophobic. That is the membrane surfaces were made hydrophilic by exposure to ultraviolet ozone activation/radiation.

### 2.2. Characterization of Silicone Membranes

#### 2.2.1. Contact Angle Measurements

Contact angle measurements on the substrate were made, on a goniometer, using the sessile drop method. 2 *μ*L droplets of water, suspended from the tip of a microliter syringe supported above the sample stage (Rame-Hart 100-00) were allowed to drop on the sample surface. Images of the droplet were captured with a CCD camera (Zoom 7000 Navitar TV Zoom). The contact angles were measured and analyzed using an ATI multimedia Player and Scion Image program (Microsoft).

#### 2.2.2. Atomic Force Microscopy

Roughness analysis of the silicone surfaces were performed using a Dimension 3000 Atomic Force Microscope (Digital Instruments, Santa Barbara, CA) under ambient conditions, on a 10 *μ*m × 10 *μ*m scan size. The mean Ra roughness (nm) of both nontreated and functionalized surfaces was measured. Topographical images were acquired in a tapping mode using silicon tips on integral cantilevers with nominal spring constant of 20–100 N/m. Images were obtained from, at least, three different samples prepared on different days and on three microscopically separate areas on each sample. AFM scans were taken on substrates to determine relative changes in surface roughness after the surfaces were modified.

#### 2.2.3. Rutherford Backscattering Spectrometry Analysis

Beams of ^4^He^++^ from the accelerator were utilized with a standard backscattering (BS) setup to obtain BS spectra of silicone membranes containing C, O, and Si. The relevant RBS detector parameters were: current = 10 nA, FWHM = 20, solid angle subtended by detector = 10°, backscattering angle = 10°, and the beam energy = 3.44 MeV. An incident monoenergetic He^++^ beam used as the incident probe was elastically scattered off by target atoms such as ^28^Si, ^8^O and ^12^C. To enhance sensitivity to light elements, beam energy of 3.44 MeV was chosen since C and O have cross-section resonance near 3.40 MeV and 3.1 MeV, respectively. Simulations were performed using RUMP^R^ (RBS Analysis and Simulation Package, version 4, 2002, Computer Graphics Service Ltd, El Passo, TX). RBS, a commonly applied technique in quantitative surface analysis with the ability to determine, at 1–3% precision, the elemental composition of samples and depth distributions was used to ascertain the surface modification reaction and to measure the depth profile of the elements Si, O, and C.

### 2.3. Fibronectin Adsorption

Both the functionalized and nontreated silicone membranes were incubated in a 2.5 *μ*g/mL concentration of FN (Sigma-Aldrich, St Louis, MO) solution for 1 h at 37°C. The FN molecules were bound to the membrane surfaces by physisorption. The non-attached FN molecules were then removed by washing with physiologic buffer, PBS (ph 7.4). Prior to cellular attachment, this concentration of 2.5 *μ*g/mL of FN was coated on the different surfaces for 1 hour incubation at 37°C in order to attain monolayer surface coverage [20].

### 2.4. Cell Culture

MC3T3-E1 cells were maintained in 22 mL of complete medium consisting of Dulbecco's Modification Eagles Medium (DMEM) supplemented with 10% fetal bovine serum, 2 mM L-glutamine and 50 *μ*g/mL penicillin/streptomycin (PSF), pH 7.0. Cells were passaged once every week and fed every other day with complete DMEM supplemented with 10% FBS. The cells were cultured at 37°C in a humidified atmosphere containing 5% CO_2_.

### 2.5. Application of Mechanical System to the Silicone Membranes

**Figure 1:**
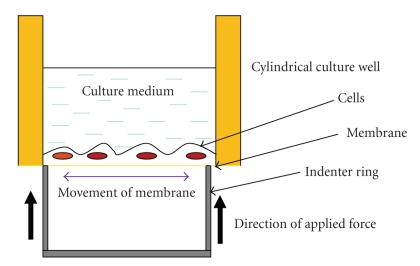
Schematic diagram showing the four-point bending principle. The principle of low strain (equibiaxial strain) cell stretching, using the four-point bending system.

The design and characterization of the system to apply mechanical forces to the cell culture consisted of an apparatus capable of deforming a compliant substrate and generating reproducible mechanical strains, mimicking *in vivo* conditions. This was to ensure the maintenance of cell viability during cell culture, attachment to the membrane and deformations that mimicked *in vivo* conditions and the possibility of imaging the system for real-time data acquisition. Controlling the cell environment meant providing conditions of 37°C, 5% CO_2_ and 100% humidity. These conditions were achieved in this experiment using a cell chamber. Application of mechanical forces to cells seeded on the membranes conformed to techniques reported by Banes and coworkers [21]. The cell stretching device that incorporated the four-point bending system was fabricated at the Department of Anatomy & Cell Biology, School of Dental Medicine, the University of Pennsylvania. The silicone membranes were attached to polystyrene cylinders with an O-ring, under sterile conditions (Figure 1). The cylinder was screwed into a base with a glass window resting on another O-ring to create a chamber. 

The apparatus designed as a four-point bending system was capable of deforming a compliant substrate in order to generate a reproducible mechanical strain. It provided a sinusoidal waveform and an equibiaxial stress within a frequency range of 0.2 Hz–2.0 Hz for a period of 2 hours under an atmosphere of 100% humidity, comparable to physiological conditions. As a result of applying loads up to 1000 Pa, to produce strain rates of up to 4%, the membranes experienced strain regimes of 200 *μ* *ε*–2500 *μ* *ε* in all four directions. The cells were seeded on the membranes at a concentration of 100,000 cells/cm^2^ for 24 hours, after which the membranes were subjected to a uniform equibiaxial strain. A 2% equibiaxial strain was applied at a frequency of 1 Hz, in this experiment, because these conditions were postulated to be physiological [22]. The experiments were terminated after 2 h and the cellular responses were evaluated. As a control, the osteoblasts cells were seeded on membranes prepared, as previously described, but not stretched.

### 2.6. Cellular Response to Mechanical Stimulus

#### 2.6.1. Cytoskeletal Analysis of Cells Attached to Si Membranes

 Actin filaments were visualized by treatment with Alexafluor 568 conjugated phalloidin (Molecular Probes, Eugene, OR, USA). The medium was removed from each sample and the cell layer was washed twice with PBS. Cells were fixed with 1.5% formalin in PBS for 5 minutes. Triton X-100 (0.1%) in PBS and 1% bovine serum albumin (BSA) were added to permeabilize the cells. After 20 minutes, the cell layer was washed twice with PBS and then incubated with phalloidin (1 : 100) in PBS with 0.1% Tween 20% and 1% BSA overnight at 4°C. Cells were analyzed with a confocal microscope (the Olympus Fluoview), inverted, with a long-working distance lens. A specialized cap was used to enable evaluation of the cells through a plastic holder. In order to quantify the cells, a plane of maximum fluorescence was determined. The photomultiplier tube voltage was set at that plane point to serve as the control well

### 2.7. Statistical Analysis

The experiments were repeated three times and similar results were obtained with each of the replicates. Data were analyzed using a one-way ANOVA where appropriate, with Scheffe's test at a level of 0.05. The differences among the cell incubation times were statistically analysed at the same level.

### 2.8. Results

#### 2.8.1. Characterization of Silicone Membrane Surfaces

**Figure 2:**
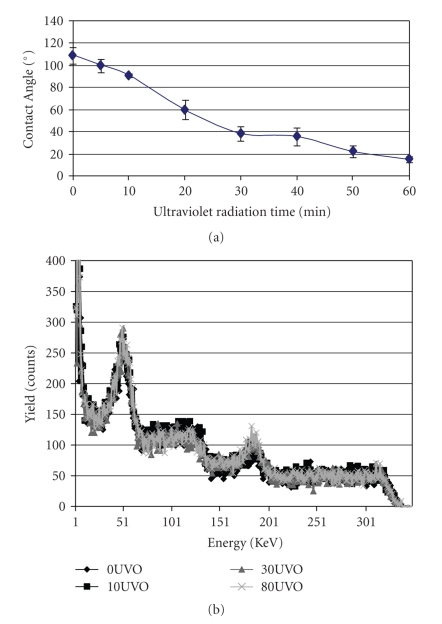
(a) Characterization of Si membrane using the water contact goniometry method. The figure shows the degree of surface wettability of Si membranes after exposure to UVO radiations at different times. (b) Characterization of Si membrane using Rutherford Backscattering spectrophotometry. Figure shows similar profiles for all the samples of Si membranes after exposure to UVO radiations at 4 different times.

The treatment procedures of the silicone membrane surface were optimised in order to maintain its elastic properties. Observations made initially indicated that there were very minimal alterations made to the physical properties of the silicone membranes. In fact, when the membranes were subjected to equibiaxial strain, there was no evidence of deformation of the substrate. Surfaces of the functionalized membrane were characterized by contact angle measurements over time (Figure 2(a)). Data showed a steady decrease in the surface wettability of the membrane. In fact, there was up to 20° reduction in surface wetting after 10 minutes UVO exposure (Figure 2(a)). Exposure of the membrane surfaces for longer time periods however, did not reduce the surface hydrophobicity of the Si membranes, further.

#### 2.8.2. Surface Roughness of the Silicone Membrane

**Figure 3:**
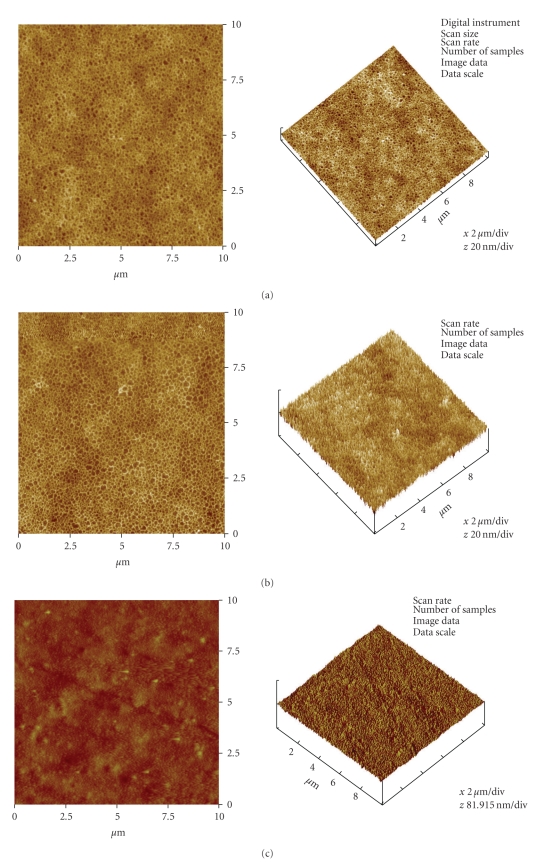

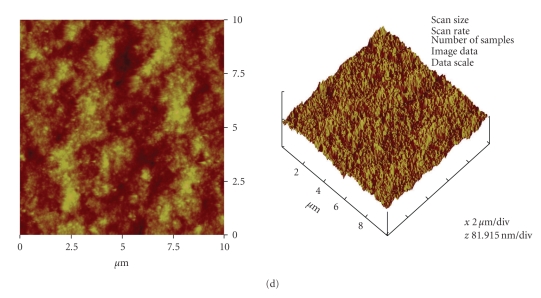
(a) Representative AFM images of a raw and unclean Si membrane surfaces. Roughness analysis done on a 10 *μ*m scan size of the images indicated that the Surface roughness (Ra) on these surfaces was 0.8 nm; while the RMS roughness value was 1.0 nm. (b) Representative AFM images of a non-UVO-activated water-cleaned Si membrane surface. Roughness analysis on a 10 *μ*m scan size of the images indicated that the Surface roughness (Ra) on these surfaces was 1.2 nm; while the RMS roughness value was 1.4 nm. (c) Representative AFM images of a 10 min UVO-activated Si membrane surface. Roughness analysis on a 10 *μ*m scan size of the images indicated that the Surface roughness (Ra) on these surfaces was 1.9 nm; while the RMS roughness value was 2.4 nm. (d) Representative AFM images of a 30 min UVO-activated Si membrane surface. Roughness analysis on a 10 *μ*m scan size of the images indicated that the Surface roughness (Ra) on these surfaces was 4.7 nm; while the RMS roughness value was 5.9 nm.

In order to characterize the topography of the silicone membrane, its surface composition was analysed using Rutherford backscattering spectrometry (RBS) while surface roughness was evaluated by means of atomic force microscopy (AFM). RBS plot in Figure 2(b)showed similarity in the surface profiles of three of the different samples of the membrane after exposure to various UVO times. AFM images (Figure 3(a) to 3(d)) showed a steady increase in the surface roughness of the membranes after 10 to 30 min UVO treatment compared to the nonactivated sample. After 10 min UVO treatment, the mean nanoscale surface roughness (RMS) was 2.4 nm (Table 1). After 30 min UVO exposure however, surface roughness was more than doubled (cf. Figure 3(d) with Figures 3(b)and 3(c)). 

**Figure 4:**
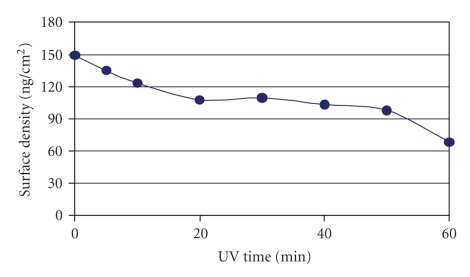
Surface density characterisation of FN-coated UVO-activated Si membrane surfaces. Up to 60 min UVO-activated surfaces were coated with 2.5 *μ*g/mL concentrations of FN. 2.5 *μ*g/mL of FN was coated on surfaces for 1 hour incubation at 37°C in order to attain monolayer surface coverage [20].

**Figure 5:**
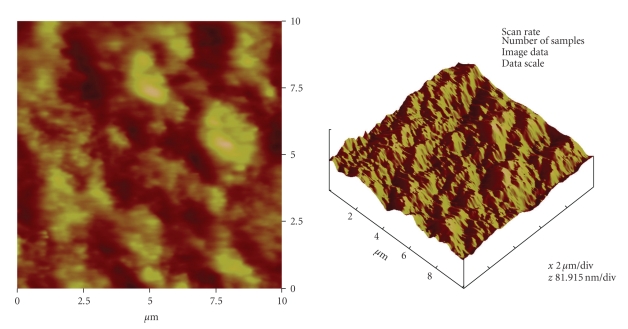
AFM images of 30 min UVO-activated Si membrane surfaces precoated with 2.5 *μ*g/mL FN. The surface roughness of (RMS) 3.7 nm was obtained. Plane view of the figure (left) shows clusters of FN molecules on the activated Si membrane; while the elevated view shows very rough ridges of clustered FN molecules adhering to the Si surfaces.

The surface density of FN molecules evaluated on the functionalized silicone surfaces indicated a nanoscale monolayer protein coverage of up to 150 ng/cm^2^ (Figure 4). There was generally, a significant decrease in roughness on all the three surfaces after adsorbing 2.5 *μ*g/mL FN onto the functionalized surfaces; and more specifically, a mean surface roughness (RMS) of 3.7 nm (Table 1) was observed on the 30 min functionalized silicone membrane surface (Figure 5).

#### 2.8.3. Cytoskeletal Organization in Response to Mechanical Forces

**Figure 6:**
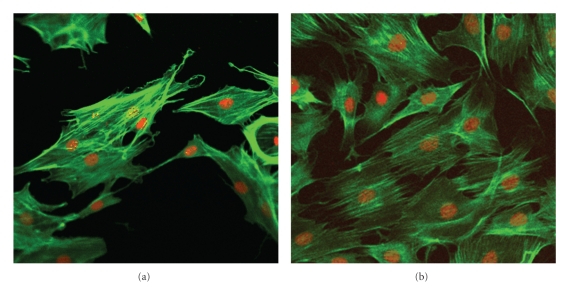
Phalloidin-stained actin filaments of osteoblasts proliferated on silicone membranes. MC3T3-E1 osteoblast cells were seeded on FN-coated silicone membrane and subjected to equibiaxial strain for 2 h. Cells were treated with rhodamine-labeled phalloidin (1 : 100) and then visualized by confocal microscopy. (a) Cells on: *nonstretched* non-UVO-activated FN-coated silicone membrane; (b) Cells on: *stretched* non-UVO-activated FN-coated silicone membrane. Note the slightly elevated fluorescence of the actin filaments in the stretched sample.

**Figure 7:**
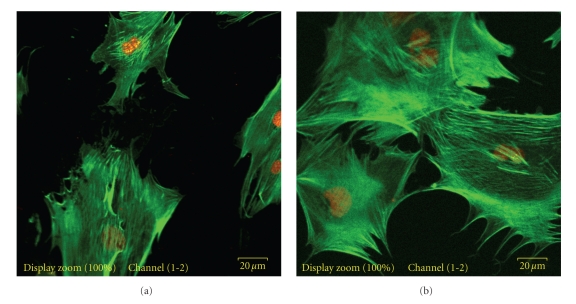
Phalloidin-stained actin filaments of osteoblasts grown on silicone membranes. MC3T3-E1 osteoblast cells were seeded on FN-coated silicone membrane and subjected to equibiaxial strain for 2 h. Cells were treated with rhodamine-labeled phalloidin (1 : 100) and then visualized by confocal microscopy. (a) Cells on: *nonstretched* UVO-activated FN-coated silicone membrane; (b) Cells on: *stretched,* 30 min UVO-activated FN-coated silicone membrane. Note the slightly elevated fluorescence of the actin filaments in the stretched sample.

**Figure 8:**
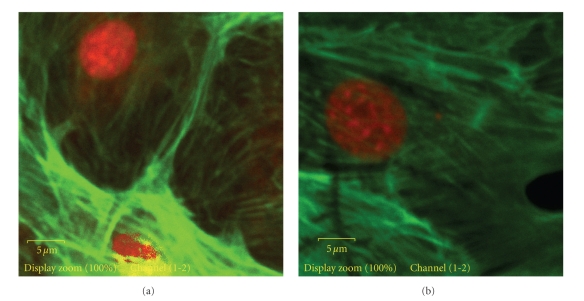
Phalloidin-stained actin filaments of osteoblasts grown on silicone membranes. MC3T3-E1 osteoblasts were seeded on FN-coated silicone membrane and subjected to equibiaxial strain for 2 h. Cells were treated with rhodamine-labeled phalloidin (1 : 100) and then visualized by confocal microscopy. Close up of: (a) Cells on *nonstretched* 30 min UVO-activated FN-coated silicone membrane; (b) Cells on *stretched* 30 min UVO-activated FN-coated silicone membrane. Note the slightly elevated fluorescence of the actin filaments in the stretched sample.

The cytoskeletal organization of cells was evaluated by staining actin filaments with phalloidin, after applying 2% dynamic equibiaxial strain exerted cyclically at 1 Hz frequency (Figures 6(a) to 8(b)). The MC3T3-E1 osteoblast cells that were subjected to strain for 2 h displayed a significant level of actin fluorescence (Figures 6(a), 7(a) and 8(a)). Although no preferential orientation was observed, there was however, a significant increase in actin filament fluorescence at the cell periphery. On the nonstretched FN-coated membrane, the actin filaments remained organized. The fluorescence intensity was generally considerably lower than the level displayed by the stretched cells (Figures 7(b) and 8(b)).

### 2.9. Discussion

The Frost mechanostatic theory assumed that the application of biophysical forces was supposed to be translated into a cellular response, thereby allowing the cellular organism to adapt to its mechanical environment. These biophysical forces involved a physiological strain range of 200 *μ* *ε* ≤ × ≤5000 *μ* *ε*. A strain regime below 200 *μ* *ε* resulted in a net bone loss; while between 200 *μ* *ε* and 2500 *μ* *ε*, the strain regimes were described as physiological. Above 5000 *μ* *ε* the strain regime was described as pathological [23]. In this and other models that have been developed to mimic the physiological environment, the mechanical stimuli were reported to occur *in vivo* and included fluid shear, hydrostatic compression, uniaxial stretch and biaxial stretch [24]. In line with the objective of this study which was to engineer a deformable silicone membrane capable of enhancing osteoblast cellular attachment and proliferation; the UVO-activated silicone membranes were first physisorbed with the extracellular matrix protein, FN, which provided ligands for integrin receptors. This functionalized silicone-biomolecule (FN) substrate promoted attachment and proliferation of the bone cells, in conformity with data [25–27] reported in previous studies. 

The data we obtained, after subjecting the osteoblast-like cells to the dynamic equibiaxial strain via the functionalized FN-coated silicone membrane, indicated that there were noticeable changes in the cytoskeletal architecture with minimum cell damage. Our data indicated that the modified silicone surfaces transduced mechanical stimuli directly to the attached cells, in conformation with a recent study that reported a direct effect of mechanical stimuli on osteoblast-like cells [28], probably through integrin receptors present in FN. The functionalized silicone surfaces created by exposure to UVO radiation, prior to attaching the FN molecules, counteracted the possibility of cell loss due to application of mechanical strain. The silicone substrate was probably bound directly to the attachment domain (RGD) of the FN molecule. 

Variations in the physical characteristics of the silicone membrane had been linked to the formation of an oxide layer after exposure of membrane to the UVO radiation, causing the formation of an excess of oxygen bridges with the functionalized silicone membrane [29]. The extent of the functionalization time was maintained at 0 to 30 min UVO exposure time, in order to preserve the elastic characteristics of the membranes. Certain factors that might have contributed to promoting the adherence of osteoblast cells to the functionalised FN-coated engineered silicone surfaces included the RGD integrin receptors present in FN molecules, which might have bound to the subunits of the osteoblast-like cells' integrin receptor [20]. In addition, one of the major factors that promoted the adhesion of osteoblasts was the modified surface topography of the silicone membrane. That is, increased surface roughness as evidenced by data obtained in this study, enhanced attachment of the osteoblast cells, as had been reported in other studies [27, 30, 31]. 

In order to assess the impact of mechanical forces on the attached bone cells, a dynamic equibiaxial tensile strain was imposed on the cellular-attached, FN-coated Si surfaces. The preliminary data obtained in this study indicated that cellular response in the physiological environment may be due to the applied equibiaxial strain to the membrane. That is, the applied strain caused the transduced forces in the membrane to be transmitted to the attached cells. There was therefore an even distribution of the dynamic equibiaxial strain across the silicone membrane which was ensured by the design of the setup [32, 33]. 

It was also evident from this study that the strain mechanisms involving a transfer of strain to the osteoblast layer tend to modulate cell function. We observed that the osteoblast cells that were subjected to equibiaxial strain displayed a significant change in morphology evidenced by slight elevation in actin filament staining (indicated by confocal microscopy images). This was in conformity with earlier reports by Toma and coworkers [34], Wang and coworkers [35] and Meazzini and coworkers [28] and a more recent data [36] which showed that cells responded to the applied force by changing their morphology. The morphological change could probably be linked to remodelling of the cytoskeletal structure of the cells. The attachment and proliferation of cells on the silicone substrate was thus enhanced, confirming the statement that applied forces to the membrane altered osteoblast cellular function. The results also suggested that the applied forces were directly transmitted to the cells via the cytoskeletal system as a result of the linkage between the osteoblast and the membrane through the Fn-integrin receptors. 

The data obtained in this study indicated that we have engineered a system that employed functionalization method to activate surfaces of silicone membranes in order to enable linkage of various biomolecules, proteins, and cells. Furthermore, bone cell adherence and proliferation were more enhanced on strained, functionalized silicone-RGD (FN molecules) surfaces. As a result of the elastic characteristics of the functionalized silicone surfaces, further mechanisms by which bone cell functions could be physiologically influenced through mechanical strains could be explored.

## Figures and Tables

**Table 1:** Table showing the RMS surface roughness values obtained by means of atomic force microscopy measurements on non-UVO/UVO-activated silicone membrane surfaces without and with, adsorbed 2.5 *μ*g/mL of, FN molecules.

UVO radiation time (min)	UVO + 0 *μ*g/mL Fn Ra (nm)	UVO + 0 *μ*g/mL Fn RMS (nm)	UVO + 2.5 *μ*g/mL Fn RMS (nm)
0	1.2	1.4	0.9
10	1.9	2.4	1.5
20	2.8	3.6	2.3
30	4.7	5.9	3.7
